# Supplementary material for: Determinants of maternity services utilisation among women of reproductive age across sub-Saharan Africa
Source: PLoS One. 2026 Feb 13;21(2):e0321521. doi: 10.1371/journal.pone.0321521 (PMC12904469; doi:10.1371/journal.pone.0321521)
Supplement: Attachment — Submitted filename: Reviewers replies_R2.pdf [file pone.0321521.s004.pdf]

Reviewers comments and replies

Reviewer #1: (No Response)

Reply

Thank you for taking the time to review our manuscript. We greatly appreciate your feedback and constructive comments.

Reviewer #3: This retrospective cross-sectional study employed Demographic Health Surveys (DHS) conducted in 27 countries in SSA. The outcome and explanatory variables were adequately defined.

Reply

Thank you for taking the time to review our manuscript. We greatly appreciate your feedback and constructive comments.

On Line 164 the authors state , ".... three-level multi-level logistic regression...." Do they actually mean, "three-level multiple logistic regression..."?

Reply

This study employed a three-level multilevel logistic regression analysis rather than a multiple regression approach, as it accounted for unexplained variations across all levels. Models 0 to IV were assessed using log-likelihood ratio and Akaike Information Criterion (AIC), as presented in Tables 3 to 5.

Tables 3,4 and 5 are well constructed as one would expect a hierarchical presentation. Since this was a multilevel look, the authors did put a descriptive paragraph at the end of the Tables 3,4, and 5 presentation stating the relative merits of the three constructs in some way. However, this could have been a bit more sophisticated beyond the model fit statistics with a Factor analysis type structure if individual, household and community separate out to three meaningful constructs to determine which grouping is the most statistically significant contributor to the three endpoints being investigated. Why , no attempt for a Data reduction strategy in view of the many variables being examined

separately? There is no explanation in the discussion section as to the statistical overall comparison of the three levels (constructs) as to their relative importance individually or why that was not considered. This could possibly be a study limitation from the analytical perspective.

Reply

Thank you for raising this important observation. We have addressed it in the limitations section of the revised manuscript. The updated Strengths and Limitations section now reads as follows:

"This study analysed pooled DHS datasets from 27 sub-Saharan African countries involving 58,648 women of reproductive age. Using nationally representative data provided a robust and comparative picture of the facilitators and barriers to maternity service utilisation across the region. The inclusion of a decade-long dataset enhances the reliability and generalisability of the findings, making an important contribution to advancing knowledge on maternal and newborn health coverage in SSA. However, the exclusion of 19 countries due to unavailable data limits full regional representation. Reliance on self-reported data introduces the possibility of recall bias, while the cross-sectional design prevents determination of causality. In addition, the absence of a data reduction strategy, such as factor analysis, restricts the ability to assess the relative contributions of individual, household, and community-level factors. Finally, given the study's scope and eligible respondents, findings may not fully align with patterns reported in other literature."
