# Supplementary material for: Determinants of maternity services utilisation among women of reproductive age across sub-Saharan Africa
Source: PLoS One. 2026 Feb 13;21(2):e0321521. doi: 10.1371/journal.pone.0321521 (PMC12904469; doi:10.1371/journal.pone.0321521)
Supplement: Attachment — Submitted filename: Comment plos.docx [file pone.0321521.s001.docx]

Comment

General comments:

--writeup in introduction is well

- Writeup in method is a litle bit good.

-result write up have good floow and detail

-the overall result will have significant impact

Spesfic comments,

1. Title:is better to be stated as Determinants of maternity services utilisation Across SubSaharan Africa
2. From line number 127 and 128, you state as you considered 58648 reproductive women from 27 country but not state how you poold different contry data.
3. Your propsed model for out come variable is good.but there are mony resaerch that have been conducted on your resaerch area using multilevel model. So is it apropriate model for your research problem? Why not count models?
4. From line number 182; you you state “you have model comparasion but you did not state for what purepose you do model comparasion.
5. From line number 251 you state “model 4” but from line number 182 you stated”model IV” , so the numbersig method is better to be the same and the same is true for onthres.
6. “You stated as model comparasion have been done” but I did not get model compasion results for three models using AIC, BIC and other you stated method.
