# Supplementary material for: Determinants of maternity services utilisation among women of reproductive age across sub-Saharan Africa
Source: PLoS One. 2026 Feb 13;21(2):e0321521. doi: 10.1371/journal.pone.0321521 (PMC12904469; doi:10.1371/journal.pone.0321521)
Supplement: Attachment — Submitted filename: Reviewers replies_R1.docx [file pone.0321521.s003.docx]

**Reviewers’ comments and replies**

**Reviewer #1**: Thank you for your valuable work on this important topic. Your research addresses a prevalent and systematic issue, and your efforts are much appreciated. Here are my comments and suggestions for improving the manuscript:

Reply

Thank you for taking the time to review our manuscript and we thank you for your review and comments that have improved our work.

**Abstract Section**
- Lines 29 and 30: "In 2020, approximately 800 women died daily as a result of largely preventable complications of pregnancy and delivery" – Please specify the location of these deaths. Is this a global statistic?

Reply

Yes, it’s a global statistic, and this has been added. Line 30 and 63.

- Lines 55 and 56: "whilst women who were gainfully employed were more likely to utilise postnatal care within 48 hours of delivery" – The term "gainfully employed" is broad and should be clearly defined in the methods section.

Reply

The word “gainfully employed” has been changed to “employed” throughout the manuscript; information about employment was explained under the methods section in line 150.

**Background Section**
- Lines 63 and 64: This should be modified in line with the first comment in the abstract section.

Reply

Yes, it’s a global statistic, and this has been added. Line 30 and 63.

- Lines 94 to 97: It would be more appropriate to include this information in the methodology section, specifically under outcome variables and explanatory variables.

Reply

Yes, we included it. Lines 135 to 142.

- Overall, try to summarise and shorten the background section. Avoid repetitive ideas and combine related sentences for better clarity.

Reply

The background section has been restructured to avoid repetitions of ideas.

Results Section
- Lines 234 and 235: "For the household wealth index, 20% of the women reported being from poorer, middle, richer, and richest households" – This sentence needs restructuring to convey a clearer message.

Reply

The paragraph has been rewritten to convey a clear message. Page 10, lines 234 to 237.

- When reporting the results of the multilevel regression analysis, mention both comparison groups. For example, on page 17, lines 278 to 280: “it can be reported that the older a woman, the more likely she was to deliver at a health facility with women aged 25-34 and 35-49 being 19% (aOR=1.18; 95% CI:1.10 -1.26) and 39% (aOR=1.39; 95% CI: 1.28 – 1.51) significantly more likely to deliver at a health facility respectively” – More likely to deliver compared to which group? Ensure the comparison group is mentioned in all relevant instances.

Reply

Thank you for the observation. This has been corrected throughout the manuscript.

Discussion Section
- The discussion should be rewritten, refined, and shortened. It should not merely repeat the results section. Instead, discuss possible reasons for the study's major findings and variations compared to other studies. Compare and contrast your results with the existing literature.

Reply

This section has been rewritten and reduced.

- For the result mentioned in lines 308 to 312, where women with primary and secondary education are more likely to have PNC compared to those with no education, discuss possible reasons for this finding.

Reply

More information about this has been provided in the discussion section in line with the study results.

- Discuss why women in polygamous marriages were less likely to have ANC visits but more likely to have PNC visits compared to those in monogamous relationships. Additionally, explore reasons why women with social media exposure, from households with higher wealth indices, and those in high literacy communities are less likely to have PNC visits.

Reply

More information about this has been provided in the discussion section in line with the study results.

Conclusion Section
- The conclusion should be rewritten and shortened. Focus on answering the objective of the study and providing clear, concise conclusions.

Reply

This section has been rewritten and reduced.

Your attention to these details will greatly enhance the clarity and impact of your manuscript. Thank you for considering these suggestions.

Reply

We have checked through the manuscript again and have made substantial changes to improve the manuscript.

**Reviewer #2: Comment**

General comments:

--writeup in introduction is well

- Writeup in method is a litle bit good.

-result write up have good floow and detail

-the overall result will have significant impact

Reply

Thank you for taking the time to review our manuscript and we thank you for your review and comments that have improved our work.

1. Title:is better to be stated as Determinants of maternity services utilisation Across SubSaharan Africa

Reply

The title has been changed to reflect this.

2. From line number 127 and 128, you stated as you considered 58648 reproductive women from 27 countries but not state how you pooled different country data.

Reply

Information about this has been added to page 6, lines 173 to 175, under statistical analyses.

3. Your propsed model for out come variable is good. but mony research that have been conducted on your research area using multilevel model. So is it apropriate model for your research problem? Why not using count models? ( needs your clear justification on it)

Reply

Thank you for calling our attention to this. Yes, we believed this is the appropriate model because of the nature of the dataset, and because we considered three outcome variables in this study, we believed it’s appropriate to consider using the same model for the three outcomes given the fact that “facility delivery” is dichotomous.

4. From line number 182; you you state “you have done model comparasion but you did not state for what purepose you do model comparasion.

Reply

This information has been added to ensure better understanding “to show which model significantly improves over others whilst ensuring the appropriate model with goodness of fit is selected.”

5. From line number 251 you state “model 4” but from line number 182 you stated”model IV” , so the numberig method is better to be the same and the same is true for onthres.

Reply

This has been changed to “model I to model IV” in the same format.

6. “You stated as model comparasion have been done” but I did not get model compasion results for three models using AIC, BIC and other you stated method.

Reply

The results were stated in the tables except for BIC. All other results have been reported in the results section, and the BIC claim has been deleted.
